# Relationship between trackmakers of the Laetoli footprints from gait synchronization

**DOI:** 10.1017/ehs.2025.10

**Published:** 2025-04-08

**Authors:** Wataru Nakahashi

**Affiliations:** Faculty of Social Sciences, Waseda University, Tokyo, Japan

**Keywords:** Laetoli footprints, *Australopithecus afarensis*, gait synchronization, live camera, sexual dimorphism

## Abstract

The parallel trails of footprints at Laetoli site G are important fossils for studying the characteristics of *Australopithecus afarensis*. However, the relationship between the trackmakers – i.e. whether it was that of an adult male–female pair or of parent–offspring – remains unclear. The footprints show that the two individuals walked side by side with a narrow and constant distance between them and synchronized their leg movements and step lengths (gait synchronization), although they had a large height difference. In this study, live camera videos were collected to obtain data on gait synchronization in *Homo sapiens*, the closest extant species to *A. afarensis*. The data showed that when two humans with a large height difference walked alongside each other, with (at least) one of the pair having their arm around the other’s shoulder or back, adult male–female pairs (couples) frequently synchronized their gait, but parent–offspring pairs did not, whereas both couples and parent–offspring seldom synchronized when they walked side by side without connection or with handholding. Two individuals only maintained a narrow and constant distance like that between the Laetoli footprints when they walked with an arm-around connection. Therefore, assuming that *A. afarensis* had the same gait synchronization tendency as *H. sapiens*, the trackmakers were more likely to be an adult male–female pair than a parent–offspring one.

## Graphical abstract

**Figure:**
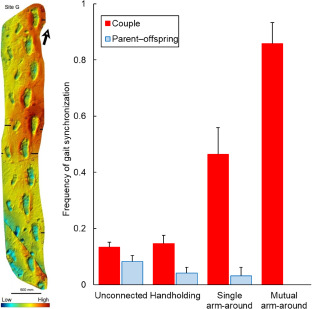

## 1. Introduction

**Figure 1.**
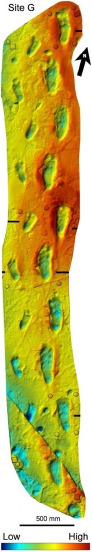
Shaded 3D photogrammetric elevation model of the footprints at the southern sector of Laetoli site G (modified from Masao et al., 2016).

The Laetoli footprints are important trace fossils for investigating the morphology and behaviour of early hominins, particularly *Australopithecus afarensis* (Getty Conservation Institute, 1996, 2000, 2001, 2011; Leakey, 1981; Leakey & Hay, 1979; Tattersall, 1998; White & Suwa, 1987). Although footprints at site A received considerable attention recently (McNutt et al., 2021), those that have attracted the most interest were found at site G, where three individuals had made parallel trails (Fig. 1). Small (G1) and large (G2) individuals walked side by side, and an intermediate-sized individual (G3) followed behind, stepping on the footprints of G2 (Getty Conservation Institute, 1996, 2000, 2001, 2011; Leakey, 1981; Tattersall, 1998). Many researchers have investigated the relationship between them on the basis of footprint shape. Focusing on the small and large individuals (because the possibility that G3 walked there some time after the two other individuals had done so cannot be excluded), some consider that they were an adult male and female pair (Tattersall, 1998), while others argue that they were an adult and a child (probably a parent–offspring pair) (Leakey, 1981; Masao et al., 2016). Considering that the footprints are not well preserved and existing foot fossils of *A. afarensis* are of poor quality and quantity, reaching a consensus is difficult. Recently, new footprints (S1 and S2) were found in Laetoli (Masao et al., 2016), which are appreciably larger than previous ones, suggesting a considerable body size variation in this species that might indicate large sexual dimorphism. However, if G1 and G2 were an adult male–female pair, this would suggest that the variation existed not only between sexes but also within males.

The parallel trails of G1 and G2 have an interesting feature: gait synchronization. That is, the two individuals synchronized their leg movements and step lengths (Leakey, 1981; Tattersall, 1998). This feature may not have been a coincidence, because G1 and G2 had a large difference in foot length, which is a good indicator of height (Jasuja et al., 1991; Tuttle, 1987; Tuttle et al., 1990; Wiseman & De Groote, 2022). Height and step length generally correlate in humans (Guest et al., 2017; Jasuja, 1993; Jasuja et al., 1997), and a newly discovered tall individual (S1), who walked alone, had a longer step length than G1 and G2 (Masao et al., 2016; Table 1), suggesting that the same correlation existed in *A. afarensis*. Moreover, the step length-to-height ratio of S1 lies between those of G1 and G2 (Table 1; calculated from Masao et al., 2016), which indicates that G2 may have shortened his/her step length, as the ratio generally decreases slightly with height in humans (Ahmedov et al., 2022). This confirms that the two individuals did not walk at different times, as G2’s shortened step length would be inexplicable if G2 had walked alone. Although some researchers have investigated gait synchronization in humans (Zivotofsky & Hausdorff, 2007; van Ulzen et al., 2008; Zivotofsky et al., 2012, 2018; Chambers et al., 2019; reviewed in Felsberg & Rhea, 2021), it is uncertain which type of pair, an adult male–female pair (couple) or a parent–offspring one, more frequently synchronize their gait if they have a large difference in height. Given that *Homo sapiens* is the closest extant species to *A. afarensis*, the characteristics of human locomotion may provide valuable insights for estimating those of *A. afarensis*.

**Table 1.** Data and estimates for G1, G2, and S1 (from Masao et al., 2016)

	G1	G2	S1
Footprint length (cm)	18.0	22.5	26.1
Step length (cm)	41.6	45.3	56.8
Stride length (cm)	82.9	88.0	113.9
Estimated height (cm)	111 − 116	139 − 145	161 − 168
Step length/Footprint length	2.31	2.01	2.18
Stride length/Footprint length	4.61	3.91	4.36
Step length/Estimated height	0.359 − 0.375	0.312 − 0.326	0.338 − 0.353
Stride length/Estimated height	0.715 − 0.747	0.607 − 0.633	0.678 − 0.707

## 2. Methods

### 2.1. Overview

In this study, live camera videos were collected from YouTube to determine the frequency of gait synchronization between human couples and between a parent and an offspring. To compare with the Laetoli footprints, it is important to investigate human walking under natural conditions. The methodology complies with the terms of service and use of YouTube, and satisfies the ethics regulations related to research with human subjects at Waseda University.

Considering that the estimated height of G1 is approximately 20% smaller than that of G2 (Leakey, 1981; Masao et al., 2016), two individuals walking side by side were sampled, with one being one to two heads shorter than the other. Additionally, given that the serial parallel trails of footprints at the southern sector of Laetoli site G consist of around 10 steps (Leakey, 1981; Getty Conservation Institute, 1996, 2000, 2001, 2011; Fig. 1), a case in which two individuals synchronized their leg movements for 10 or more steps was defined as gait synchronization. That is, starting from the moment when two individuals stepped (almost) simultaneously, if an individual with a shorter step length (typically a smaller individual) had not started their eleventh step (toe-off) by the time the other (typically a larger individual) had finished their tenth step (heel-contact), their gaits were considered synchronized. In fact, most unsynchronized dyads differed in their step counts by around five steps, while most synchronized dyads continued synchronization for more than 10 steps; therefore, their categorization was straightforward. Note that 10-step synchronization is the minimum criterion for gait synchronization, although the methods and criteria used to identify it vary across previous studies, which have often analysed the gaits of dyads of similar heights under controlled laboratory conditions over longer distances (reviewed in Felsberg & Rhea, 2021). It is reasonable to apply the most lenient criteria in this study – which observed dyads with a large height difference under natural conditions – where their walking patterns were not perfectly consistent or straight. Moreover, since YouTube videos were used in this study, the more precise methods employed in previous studies, such as 3D motion capture, were unavailable. However, this was not a significant issue, as synchronized and unsynchronized dyads were generally easily distinguishable, probably because dyads with a large height difference were specifically sampled. Note that quantifying the phase synchronization of gait rhythms reveals a bimodal distribution, indicating that dyad gaits are classified as either synchronized or unsynchronized (Zivotofsky et al., 2012). Additionally, although the synchronization pattern of dyads may have differed outside the frame, considering that the Laetoli footprints represent nearly a 10-step snapshot, sampling 10-step snapshots from the webcam provides a reasonable comparison.

The contact between two individuals was categorized into three types: unconnected, handholding, and arm-around. Handholding includes cases where one individual grasps the hand or arm of the other, and the two individuals walk arm in arm. Arm-around includes cases where at least one individual places an arm around the other’s shoulder or back. If a smaller individual was a child, the pair was considered as a parent–offspring pair, and if she was an adult female, they were a couple. However, this assumption does not matter because what should be investigated is whether the small individual of the Laetoli footprints (G1) was a child. Since live camera videos taken at the beach in summer were used, many couples and families who were lightly dressed and walked with sandals or barefoot were included in the footage. Therefore, their ages, sexes, and heights were easily estimated. Note that errors in the estimation of age and sex do not affect the significant results, as they reduce the differences between categories.

### 2.2. Sample collection

Live camera videos were collected from the YouTube account ‘Beaches Be Trippin’ (https://www.youtube.com/@BeachesBeTrippin) in Ocean City, Maryland, USA, in July 2023. Weekend videos were primarily used to collect samples effectively. To obtain a sufficient sample size, videos of walking couples were collected for one day (around 12 hours), those of parent–offspring pairs for two days, and dyads with an arm-around connection for 10 days. The detailed dates and URLs of videos are shown in Supplementary Table S1. The recorded data include the relationship between two individuals (couple/father–son/father–daughter/mother–son/mother–daughter), the walking side of the taller individual (right/left), the contact between the two individuals (unconnected/handholding/arm-around (single/mutual)), gait synchronization (unsynchronized/synchronized), and the phase of synchronization (in-phase/anti-phase) if synchronized. In all samples, the taller individuals were males or parents, probably because dyads with a height difference of one to two heads were sampled. When individuals changed walking sides or the type of contact, or the phase of synchronization in the videos changed, the more clearly observable (closer to the camera) or smaller sample data were used. Considering that distinguishing between all individuals is impossible, all applicable samples were counted if they satisfied the following regulations, although some of them seemed to include the same individuals. If they were excluded, an unintentional bias may have arisen, because individuals with gait synchronization tend to be observed more carefully.

### 2.3. Sampling regulations

Videos of two individuals with a height difference of one to two heads walking side by side were collected. Since gait synchronization was defined as the synchronization of leg movements for 10 or more steps, dyads without observable 10 steps (e.g. due to walking behind others) or those who frequently changed positions and did not walk side by side for 10 steps were excluded. The following individuals were also excluded: jogging individuals, individuals carrying a pushchair, and more than two individuals walking side by side without connection.

### 2.4. Statistical analysis

To calculate the frequency of gait synchronization (number of synchronized dyads/total number) in each category, the samples that did or did not satisfy the gait synchronization criteria were counted (Supplementary Tables S2–S12). Pearson’s chi-squared test was primarily used to calculate the *P*-values of the data, but Fisher’s exact test was used when one or more expected values were below five. When two frequencies were significantly different, asterisks (*, **, ***) were used in the figures to indicate *P* < 0.05, *P* < 0.01, and *P* < 0.001, respectively.

## 3. Results

**Figure 2.**
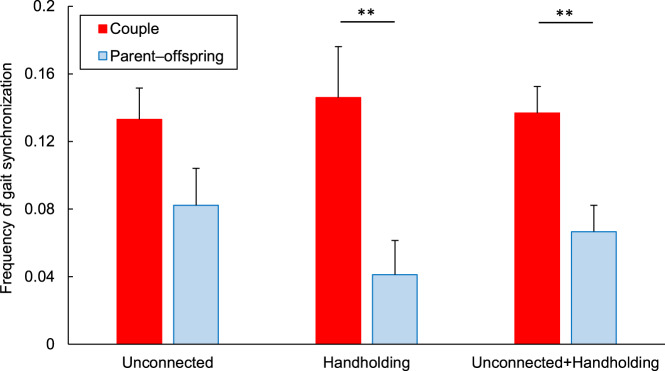
Frequencies of gait synchronization for unconnected dyads, handholding dyads, and the combined group (mean + SEM). **P < 0.01.

The data showed that the frequency of gait synchronization between couples was not significantly different from that between parent and offspring if unconnected (*P* = 0.10, chi-squared test), but higher if handholding (*P* < 0.01, chi-squared test, Fig. 2). In addition, both between couples and between parent and offspring, the frequency was not significantly different between unconnected and handholding (*P* = 0.71 and *P* = 0.20, respectively, chi-squared test). Pooling unconnected and handholding, the frequency of gait synchronization between couples was higher than that between parent and offspring (*P* < 0.01, chi-squared test). The following data were pooled in analyses because no significant differences in relation to gait synchronization were observed (Supplementary Tables S2–S12): the walking side of the taller individual (right/left), the sexes of parent–offspring (father–son/father–daughter/mother–son/mother–daughter), and the phase of synchronization (in-phase/anti-phase). Despite the lenient gait synchronization criteria in this study, the frequency of gait synchronization was lower than in previous studies (Chambers et al., 2019; van Ulzen et al., 2008; Zivotofsky et al., 2012, 2018; Zivotofsky & Hausdorff, 2007), probably because only dyads with a large height difference under natural conditions were sampled. This may justify the use of the lenient criteria in this study.

Despite significant differences in the frequency of gait synchronization between couples and parent–offspring, they may have been due to differences in height between the two individuals. That is, although the sampled dyads had a height difference of one to two heads, the average difference between couples may have been smaller than that between parent and offspring because lower height differences were more common in couples, whereas this tendency was not observed in parent–offspring. Therefore, no essential differences in the frequency of gait synchronization were found between couples and parent–offspring, as long as they walked side by side without connection or with handholding.

**Figure 3.**
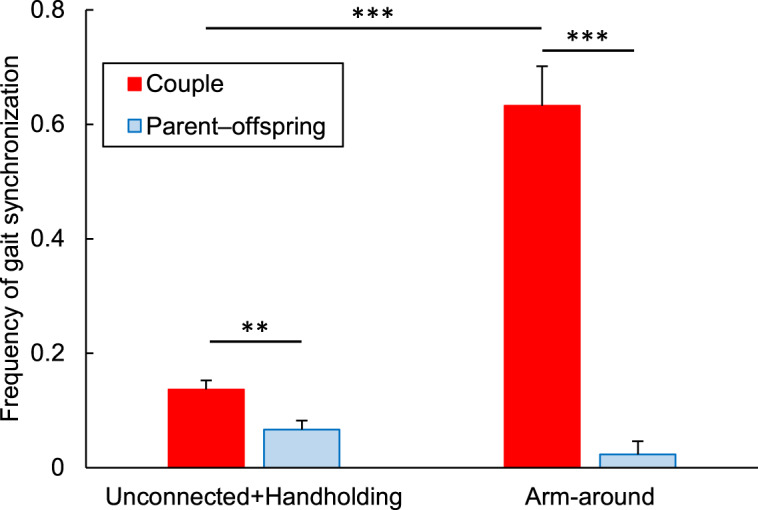
Frequencies of gait synchronization for unconnected or handholding dyads and arm-around dyads (mean + SEM). **P < 0.01, ***P < 0.001.

Compared to the frequency of gait synchronization in unconnected or handholding dyads, that in arm-around dyads was significantly higher in couples (*P* < 0.001, chi-squared test), but no significant difference was found in parent–offspring (*P* = 0.49, Fisher’s exact test, Fig. 3). The difference between couples and parent–offspring was large (*P* < 0.001, chi-squared test); i.e. the frequency of gait synchronization between couples was more than 10 times higher than that between parent and offspring. In other words, couples frequently synchronized their gait if one placed an arm around the other, whereas parent–offspring did not.

**Figure 4.**
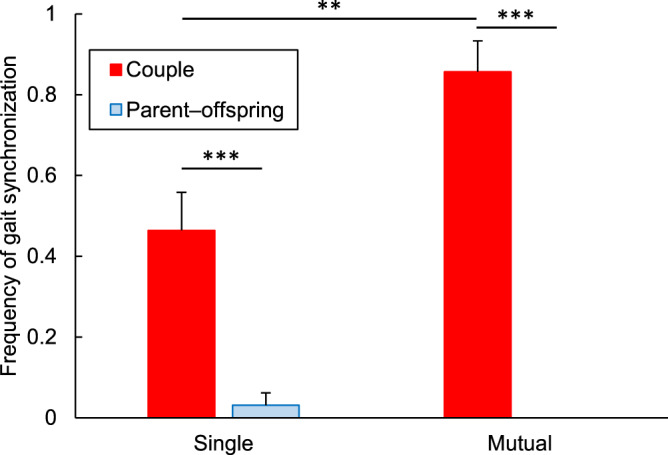
Frequencies of gait synchronization for single arm-around dyads and mutual arm-around dyads (mean + SEM). **P < 0.01, ***P < 0.001.

This relationship might stem from the differing reasons for the arm-around connection between couples and parent–offspring. Specifically, an arm-around walk between couples generally expresses deep affection, whereas in parent–offspring, it sometimes implies that the parent is guiding the child to prevent their walking in the wrong direction. In investigating this influence, arm-around dyads were categorized into two types: single and mutual. Mutual arm-around corresponds to cases in which both individuals place an arm around the other’s shoulder or back, while single arm-around indicates that only one individual places an arm around the other. Children who want to walk freely are unlikely to place their arm around their parents, so a mutual arm-around implies deep affection, whether between couples or parent–offspring. The data showed that regardless of the type of arm-around, the frequency of gait synchronization between couples was significantly higher than that between parent and offspring (*P* < 0.001 in both types, chi-squared test, Fig. 4). Therefore, the hypothesis that the differing reasons for the arm-around connection caused the large difference in the frequency of gait synchronization between couples and parent–offspring was not supported. However, the frequency of gait synchronization between single arm-around couples was significantly lower than that between mutual arm-around couples (*P* < 0.01, chi-squared test) but higher than that between unconnected or handholding couples (*P* < 0.001, chi-squared test). In contrast, the frequency of gait synchronization between parent and offspring did not differ across unconnected, handholding, single, and mutual arm-around conditions (*P* = 0.55, Fisher’s exact test). This implies that closer contact enhances gait synchronization between couples but not between parent and offspring. Note that the possibility that affectionate couples tend to prefer close contact and to synchronize their gait cannot be excluded. In fact, a few couples initiated an arm-around connection during the walk, but they synchronized their gait beforehand.

## 4. Discussion

Previous studies have shown that gait synchronization frequently occurs when tactile feedback is presented (Zivotofsky et al., 2012; Zivotofsky & Hausdorff, 2007) and when individuals are not paying attention to other tasks (Zivotofsky et al., 2018), which is consistent with the result between couples. However, these tendencies were not observed between parent and offspring under natural conditions. Although determining the reason for this observation is difficult, several hypotheses can be proposed. For example, parents may hold their children more tightly if they are more restless, but such children may also walk independently, preventing gait synchronization. Additionally, parents may have extensive experience of walking side by side with their young children, who have extremely short step lengths. As a result, they become accustomed to unsynchronized gaits and continue this behaviour even after their children have grown. In any case, provided that the difference in gait synchronization tendencies between couples and parent–offspring is not due to a human-specific factor, *A. afarensis* could be considered to have exhibited the same distinction as *H. sapiens*.

Let us look at the Laetoli footprints at site G again (Fig. 1). The distance between two individuals (G1 and G2) is quite narrow (approximately 30 cm) and remains constant throughout the trails (Getty Conservation Institute, 1996, 2000, 2001, 2011; Leakey, 1981; Masao et al., 2016; Tattersall, 1998). Considering the body size of *A. afarensis*, almost no space was found between two individuals (Leakey, 1981; Tattersall, 1998). When humans walk side by side without connection or with handholding, they must maintain a comfortable distance, which often changes because humans do not walk in a straight line under natural conditions. Therefore, two individuals can maintain a narrow and constant distance only when walking with an arm-around connection. These observations strongly suggest that G1 and/or G2 placed an arm around the other’s shoulder or back. Note that the constant distance between G1 and G2 further confirms that they did not walk at different times. This study shows that when two humans walk side by side with an arm-around connection, couples frequently synchronize their gait, while parent–offspring pairs rarely do so under natural conditions. Therefore, discovering human footprints resembling those at Laetoli site G might indicate the presence of a couple rather than a parent–offspring. The odd ratio would be more than 10-fold, although it depends on the frequency of parent–offspring pairs walking side by side with the arm-around connection relative to that of couples. Although the site observed in this study may not represent human society, the number of couples who left footprints similar to those at Laetoli site G (i.e. a narrow and constant distance between two individuals (arm-around) with in-phase gait synchronization) was 20 times larger than that of parent–offspring.

Given that the society of *A. afarensis* is not well understood, estimating the frequency of adult male–female pairs and parent–offspring walking side by side with an arm-around connection is difficult. However, humans invest significantly larger effort in child-rearing compared to other primates due to their long childhoods. Therefore, the frequency of parent–offspring walking side by side relative to that of couples would not be lower than that in other primates. Considering that the childhood of *A. afarensis* was shorter than that of *H. sapiens* (Dean et al., 2001; Smith, 1994), the relative frequency of parent–offspring pairs walking side by side with an arm-around connection in *A. afarensis* could not be considered high compared to that in humans. Therefore, if *A. afarensis* had the same gait-synchronization tendency as *H. sapiens* (i.e. when walking with the arm-around connection, adult male–female pairs frequently synchronize their gait, while parent–offspring seldom do), the Laetoli footprints at site G would be highly likely to belong to an adult male–female pair rather than a parent–offspring one.

The conclusion that an adult male–female pair left the footprints at Laetoli site G may suggest a large body-size variation among *A. afarensis* males, as S1 was significantly larger than G2 (Masao et al., 2016). This suggestion is interesting because some primate males are dimorphic (e.g. orangutans), but the possibility that S1 was exceptionally large cannot be excluded. More samples are needed to discuss this problem. Additionally, the conclusion may indicate large sexual dimorphism in *A. afarensis*, but it is important to consider the small sample size of Laetoli trackmakers, which consists of only five. Therefore, sexual dimorphism in *A. afarensis* should be comprehensively investigated by considering other fossil evidence (Grabowski et al., 2015; Plavcan et al., 2005; Reno et al., 2003, 2005, 2010). Sexual dimorphism often reflects the mating system of the species, where large sexual dimorphism is assumed to imply polygyny (Leutenegger, 1978; Weckerly, 1998). However, sexual dimorphism is not the only factor that indicates the mating system of early hominins (Lovejoy, 2009; Nakahashi, 2016; Nakahashi & Horiuchi, 2012; Nakahashi et al., 2018). When studying the mating system in *A. afarensis*, it is important to focus on their behaviour, in that an adult male–female pair walked side by side with an arm-around connection and synchronized their gait. Moreover, the conclusion of this study may offer valuable insights for morphological studies of the Laetoli footprints. For example, some researchers have analysed 3D imaging of the trails to investigate whether their bipedal gait was kinematically similar to modern humans (Crompton et al., 2012; Hatala et al., 2016; Raichlen et al., 2010). However, considering that two individuals walked with one placing an arm around the other’s shoulder or back, the weight distribution of their legs may have differed from that of individuals walking alone. This perspective may also be important when discussing the significant asymmetry of foot angles in the G1 trail, which was previously thought to have been caused by trauma or disease in his/her right lower limb (Tuttle, 1987; Tuttle et al., 1990). In any case, given that two individuals synchronized their leg movements and step lengths, direct comparisons of their footprints with those of humans walking alone are inappropriate.

To sum up, the trails at Laetoli site G show that two individuals synchronized their leg movements and step lengths while maintaining a narrow and constant distance between them. This feature suggests that they walked with one individual having an arm around the other’s shoulder or back. Observations of human dyads walking side by side indicate that gait synchronization occurs far more frequently between couples than between parent and offspring when one places an arm around the other. Applying this tendency to *A. afarensis*, it is likely that the Laetoli footprints at site G were left by an adult male–female pair rather than by a parent–offspring one.

## Supporting information

Nakahashi supplementary material Nakahashi supplementary material

## Supplementary material

The supplementary material for this article can be found at https://doi.org/10.1017/ehs.2025.10.

## Research transparency and reproducibility interest

All data used in this study are provided within the manuscript or supplementary file.
