# Supplementary material for: Relationship between trackmakers of the Laetoli footprints from gait synchronization
Source: Evol Hum Sci. 2025 Apr 8;7:e13. doi: 10.1017/ehs.2025.10 (PMC12034493; doi:10.1017/ehs.2025.10)
Supplement: Nakahashi supplementary material [file S2513843X25000106sup001.docx]

**Supplementary Table S1**

Dates and URLs of samples.

| Sample | Date | URL |
| --- | --- | --- |
| AA | July 1, 2023 | https://www.youtube.com/watch?v=KATu1NassdI |
| AA | July 2, 2023 | https://www.youtube.com/watch?v=qz90fD2cDpc |
| AA | July 8, 2023 | https://www.youtube.com/watch?v=Uoz7jssQ4dQ |
| AA | July 15, 2023 | https://www.youtube.com/watch?v=5NKMdLGI_ZE |
| AA | July 16, 2023 | https://www.youtube.com/watch?v=cnO5IoJdPNc |
| AA, C, PO | July 22, 2023 | https://www.youtube.com/watch?v=fDEDgLHBVoU |
| AA, PO | July 23, 2023 | https://www.youtube.com/watch?v=ocN9A3qWruY |
| AA | July 24, 2023 | https://www.youtube.com/watch?v=FC4LN8hdTZY |
| AA | July 29, 2023 | https://www.youtube.com/watch?v=-Av38fD9x_Q |
| AA | July 30, 2023 | https://www.youtube.com/watch?v=a3ouJLWAVBU |

Abbreviations: C = couple; PO = parent–offspring; AA = arm-around.**Supplementary Table S2**

Number of samples.

|  | Unconnected | Handholding | Arm-around | Total |
| --- | --- | --- | --- | --- |
| Couple | 338 | 137 | 49 | 524 |
| Parent–offspring | 158 | 97 | 43 | 298 |
| Father–son | 48 | 13 | 10 | 71 |
| Father–daughter | 28 | 12 | 6 | 46 |
| Mother–son | 25 | 18 | 14 | 57 |
| Mother–daughter | 57 | 54 | 13 | 124 |

**Supplementary Table S3**

Number of samples with gait synchronization.

|  | Unconnected | Handholding | Arm-around | Total |
| --- | --- | --- | --- | --- |
| Couple | 45 | 20 | 31 | 96 |
| Parent–offspring | 13 | 4 | 1 | 18 |
| Father–son | 5 | 1 | 0 | 6 |
| Father–daughter | 2 | 1 | 0 | 3 |
| Mother–son | 2 | 0 | 0 | 2 |
| Mother–daughter | 4 | 2 | 1 | 7 |

**Supplementary Table S4**

Number of samples with inphase gait synchronization.

|  | Unconnected | Handholding | Arm-around | Total |
| --- | --- | --- | --- | --- |
| Couple | 25 | 10 | 20 | 55 |
| Parent–offspring | 6 | 2 | 1 | 9 |
| Father–son | 1 | 0 | 0 | 1 |
| Father–daughter | 2 | 1 | 0 | 3 |
| Mother–son | 0 | 0 | 0 | 0 |
| Mother–daughter | 3 | 1 | 1 | 5 |

**Supplementary Table S5**

Number of samples with antiphase gait synchronization.

|  | Unconnected | Handholding | Arm-around | Total |
| --- | --- | --- | --- | --- |
| Couple | 20 | 10 | 11 | 41 |
| Parent–offspring | 7 | 2 | 0 | 9 |
| Father–son | 4 | 1 | 0 | 5 |
| Father–daughter | 0 | 0 | 0 | 0 |
| Mother–son | 2 | 0 | 0 | 2 |
| Mother–daughter | 1 | 1 | 0 | 2 |

**Supplementary Table S6**

Number of samples (taller: right).

|  | Unconnected | Handholding | Arm-around | Total |
| --- | --- | --- | --- | --- |
| Couple | 162 | 59 | 18 | 239 |
| Parent–offspring | 63 | 46 | 15 | 124 |
| Father–son | 15 | 7 | 5 | 27 |
| Father–daughter | 9 | 8 | 1 | 18 |
| Mother–son | 9 | 9 | 4 | 22 |
| Mother–daughter | 30 | 22 | 5 | 57 |

**Supplementary Table S7**

Number of samples with gait synchronization (taller: right).

|  | Unconnected | Handholding | Arm-around | Total |
| --- | --- | --- | --- | --- |
| Couple | 23 | 11 | 8 | 42 |
| Parent–offspring | 8 | 2 | 1 | 11 |
| Father–son | 1 | 0 | 0 | 1 |
| Father–daughter | 2 | 1 | 0 | 3 |
| Mother–son | 1 | 0 | 0 | 1 |
| Mother–daughter | 4 | 1 | 1 | 6 |

**Supplementary Table S8**

Number of samples (taller: left).

|  | Unconnected | Handholding | Arm-around | Total |
| --- | --- | --- | --- | --- |
| Couple | 176 | 78 | 31 | 285 |
| Parent–offspring | 95 | 51 | 28 | 174 |
| Father–son | 33 | 6 | 5 | 44 |
| Father–daughter | 19 | 4 | 5 | 28 |
| Mother–son | 16 | 9 | 10 | 35 |
| Mother–daughter | 27 | 32 | 8 | 67 |

**Supplementary Table S9**

Number of samples with gait synchronization (taller: left).

|  | Unconnected | Handholding | Arm-around | Total |
| --- | --- | --- | --- | --- |
| Couple | 22 | 9 | 23 | 54 |
| Parent–offspring | 5 | 2 | 0 | 7 |
| Father–son | 4 | 1 | 0 | 5 |
| Father–daughter | 0 | 0 | 0 | 0 |
| Mother–son | 1 | 0 | 0 | 1 |
| Mother–daughter | 0 | 1 | 0 | 1 |

**Supplementary Table S10**

Number of samples of arm-around dyads.

|  | Single | Mutual | Total |
| --- | --- | --- | --- |
| Couple | 28 | 21 | 49 |
| Parent–offspring | 32 | 11 | 43 |
| Father–son | 7 | 3 | 10 |
| Father–daughter | 4 | 2 | 6 |
| Mother–son | 11 | 3 | 14 |
| Mother–daughter | 10 | 3 | 13 |

**Supplementary Table S11**

Number of samples of arm-around dyads with gait synchronization.

|  | Single | Mutual | Total |
| --- | --- | --- | --- |
| Couple | 13 | 18 | 31 |
| Parent–offspring | 1 | 0 | 1 |
| Father–son | 0 | 0 | 0 |
| Father–daughter | 0 | 0 | 0 |
| Mother–son | 0 | 0 | 0 |
| Mother–daughter | 1 | 0 | 1 |

**Supplementary Table S12**

Frequency of gait synchronization.

|  | Unconnected | Handholding | Arm-around | Single | Mutual |
| --- | --- | --- | --- | --- | --- |
| Couple | 0.133 | 0.146 | 0.633 | 0.464 | 0.857 |
| Parent–offspring | 0.082 | 0.041 | 0.023 | 0.031 | 0.000 |
| Father–son | 0.104 | 0.077 | 0.000 | 0.000 | 0.000 |
| Father–daughter | 0.071 | 0.083 | 0.000 | 0.000 | 0.000 |
| Mother–son | 0.080 | 0.000 | 0.000 | 0.000 | 0.000 |
| Mother–daughter | 0.070 | 0.037 | 0.077 | 0.100 | 0.000 |
